# Interferon-Gamma Release Assays for the Diagnosis of Active Tuberculosis in HIV-Infected Patients: A Systematic Review and Meta-Analysis

**DOI:** 10.1371/journal.pone.0026827

**Published:** 2011-11-01

**Authors:** Jun Chen, Renfang Zhang, Jiangrong Wang, Li Liu, Yufang Zheng, Yinzhong Shen, Tangkai Qi, Hongzhou Lu

**Affiliations:** 1 Department of Infectious Diseases, Shanghai Public Health Clinical Center affiliated to Fudan University, Shanghai, China; 2 Department of Infectious Diseases, HuaShan Hospital, Fudan University, Shanghai, China; 3 Department of Internal Medicine, Shanghai Medical College, Fudan University, Shanghai, China; Vanderbilt University, United States of America

## Abstract

**Background:**

Interferon-gamma release assays (IGRAs) have provided a new method for the diagnosis of *Mycobacterium tuberculosis* infection. However, the role of IGRAs for the diagnosis of active tuberculosis (TB), especially in HIV-infected patients remains unclear.

**Methods:**

We searched PubMed, EMBASE and Cochrane databases to identify studies published in January 2001–July 2011 that evaluated the evidence of using QuantiFERON-TB Gold in-tube (QFT-GIT) and T-SPOT.TB (T-SPOT) on blood for the diagnosis of active TB in HIV-infected patients.

**Results:**

The search identified 16 eligible studies that included 2801 HIV-infected individuals (637 culture confirmed TB cases). The pooled sensitivity for the diagnosis of active TB was 76.7% (95%CI, 71.6–80.5%) and 77.4% (95%CI, 71.4–82.6%) for QFT-GIT and T-SPOT, respectively, while the specificity was 76.1% (95%CI, 74.0–78.0%) and 63.1% (95%CI, 57.6–68.3%) after excluding the indeterminate results. Studies conducted in low/middle income countries showed slightly lower sensitivity and specificity when compared to that in high-income countries. The proportion of indeterminate results was as high as 10% (95%CI, 8.8–11.3%) and 13.2% (95%CI, 10.6–16.0%) for QFT-GIT and T-SPOT, respectively.

**Conclusion:**

IGRAs in their current formulations have limited accuracy in diagnosing active TB in HIV-infected patients, and should not be used alone to rule out or rule in active TB cases in HIV-infected patients. Further modification is needed to improve their accuracy.

## Introduction

Tuberculosis (TB) is the most prevalent opportunistic infection disease and the leading killer in HIV-infected patients. In 2009, there were an estimated 1.1 million new TB incident cases and 0.4 million TB deaths among HIV positive patients, respectively [1].

Early diagnosis of TB in HIV-infected patients is of great importance. However, the gold standard for TB diagnosis remains the detection of *Mycobacterium tuberculosis* by culture, which is time consuming. Although the presence of acid-fast bacilli (AFB) in smear result in any specimen (sputum, needle aspirate, tissue biopsy) represents some form of mycobacterial disease but does not always represent TB. Moreover, diagnosis and treatment decisions may be difficult in cases with clinical suspicion of TB and negative AFB smear result in any specimen, as smear-positivity can be as low as 20% in HIV-infected patients [2]. In addition, the clinical and radiographical signs are often atypical, which further hampers TB diagnosis [3], [4], [5], [6].

The development of interferon gamma release assays (IGRAs) offered a new tool for the diagnosis of *M.tuberculosis* infection. IGRAs is based on the in vitro stimulation of peripheral blood T-cells specific using the *M.tuberculosis*-specific antigens early secretory antigenic target (ESAT)-6 and culture filtrate protein (CFP)-10 which is encoded within the region of difference-1, a region of genome absent in all Bacille de Calmette et Guérin (BCG) strains and in most nontuberculous mycobacteria [7].The presence of reactive T-cells is assessed by the induction of interferon (IFN)-gamma. There are two commercially available IGRAs currently - the ELISA-based QuantiFERON-TB Gold In Tube test (QFT-GIT; Cellestis Limited, Australia) and the ELISPOT-based T-SPOT.TB test (T-SPOT; Oxford Immunotec, Abingdon, UK).

Since IGRAs can not distinguish active TB from latent TB infection, and their performance might be impacted by the immunosuppression in HIV infected patients, their roles in diagnosis of active TB in HIV infected patients are still not established. Based on the available data from general patients and limited data from HIV-infected patients, many national guidelines have argued against the use of IGRAs for diagnosing active TB in HIV infected patients [8], [9]. However, many studies still use IGRAs for this purpose, and some of them recommended their use for active TB diagnosis in HIV infected patients [10], [11]. The newest systematic review from general patients has showed their pooled moderate sensitivity and low specificity in diagnosis of active TB (81% and 79% for QFT-GIT, 92% and 59% for T-SPOT in confirmed TB cases, respectively) after excluded indeterminate results. However, the performances of IGRAs in diagnosis of active TB among HIV-infected individuals were not specifically assessed [12].

Therefore, we conducted a systematic review and a meta-analysis in order to support the development of evidence-based guidance on the use of IGRAs for the diagnosis of active TB in HIV-infected patients.

## Materials and Methods

This systematic review was conducted according to the guidelines of the preferred reporting items for systematic reviews and meta-analyses (PRISMA) [13]. We prospectively reregistered our system review at PROSPERO. (Registration number: CRD42011001455). The protocol for this meta-analysis supporting checklist and flow diagram are available as supporting information; see Checklist S1, Protocol S1 and Flow Diagram S1, respectively.

### Search strategy

We identified studies that showed the evidence of using IGRAs in order to diagnose active TB in HIV-infected patients. We searched PubMed, EMBASE and the Cochrane-controlled central register of controlled trials and included reports from January 2001 to July 2011. Combinations of the following search terms were applied: “tuberculosis”, “T-spot”, “Quantiferon”, “interferon-gamma release assay*”, “IGRA*”, “ESAT-6”, “CFP-10”, “HIV” and “human immunodeficiency syndrome”. We also hand searched reviews and guidelines, screened citations of all included studies for additional references.

### Study selection

We included all studies that reported the assessment of two commercially available IGRAs, QFT-GIT and T-SPOT performed on blood sample to diagnose active TB. The following types of studies were excluded: 1) studies where active tuberculosis was not confirmed by M. tuberculosis culture or characteristic histopathological findings (in mixed studies, i.e. those without these strict criteria, data were analyzed for the confirmed cases separately. However, if the number of unconfirmed and confirmed patients was not presented separately, those studies were also rejected to avoid selection bias.); 2) studies performed with assays other than QFT-GIT or T-SPOT (in mixed studies, data were analyzed for the eligible cases separately); 3) studies performed on non-blood samples; 4)studies not performed and/or interpreted according to manufacturers' instructions; 5)studies including fewer than 10 HIV-infected individuals; 6) studies where selected patients were treated for TB; 7) conference abstracts, letters without original data, case reports, editorials, and reviews. Citations were independently screened by two investigators by examining titles and abstracts to identify potentially relevant studies, and differences were resolved by consensus. These original articles were then retrieved and the full text screened for final inclusion and data extraction.

### Data extraction and Study quality assessment

Data were independently extracted by two reviewers and then crosschecked. In cases of deviations, final documentation of data was based on consensus. The following data were extracted: calendar period of the study, country in which the study was conducted, sex distribution, enrolled age groups, BCG vaccination status, proportion of indeterminate results, CD4 T cells count, antiretroviral therapy status. Country was classified according to the World Bank country income classification (low/middle-income and high-income), a surrogate for TB incidence (Available at: http://data.worldbank.org/about/country-classifications/country-and-lending-groups). Additional information not specified in the original article was obtained by personal correspondence with the authors of the articles if necessary. The quality of all selected study was assessed using the QUADAS (Quality Assessment of Diagnostic Accuracy Studies) checklist, a validated tool for diagnostic accuracy studies [14], [15].

### Statistical analysis

For each study, we calculated sensitivity or specificity (and 95% CIs) and summarized the results in forest plots. The sensitivity and specificity were recalculated using data from the original papers. Only culture-confirmed cases were deemed active TB, other patients were all treated as non-active TB. Specificity data in these studies were derived from non-active TB patients who were evaluated for active TB. A random-effects meta-analysis was performed in order to account for the expected between study variability for each study, along with a pooled estimate using the MetaDisc software, version 1.4 [16]. Presence of statistically significant heterogeneity across studies was evaluated by using the chi-square test for heterogeneity in order to highlight the effect of true variability rather than sampling error on the overall variation in diagnostic estimates.

## Results

### Literature search and study selection

**Figure 1:**
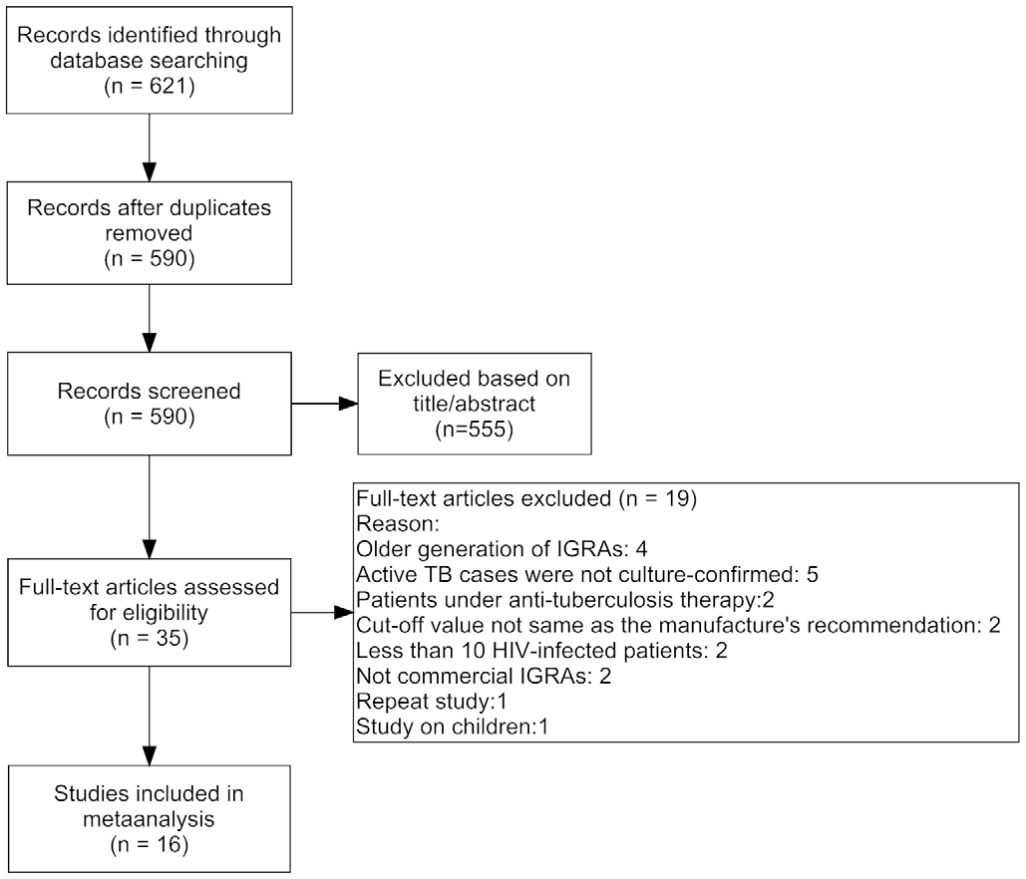
Flow diagram for study selection. IRGAs: Interferon gamma release assays.

A total of 807 studies were screened for analysis of patients with active TB using the newest two types of IGRAs from blood in HIV-infected patients. After full-text review, 16 studies included a total of 2801 HIV-infected patients (637 HIV-infected with culture confirmed TB cases) met the inclusion criteria (fig. 1).

### Characters of the selected studies

Of the 16 selected studies, 12 evaluated QFT-GIT while 6 used T-SPOT (2 studies included both of them) [10], [11], [17], [18], [19], [20], [21], [22], [23], [24], [25], [26], [27], [28], [29], [30]. The median (range) sample size was 67 (13–830) individuals enrolled per study. The studies included in the analysis were conducted in 10 different countries, most of which (70%, 7/10) are low/middle-income countries. Only 3 high-income countries (Italy, Austria and Spain) were involved in 4 studies. There are considerably variances among the studies selected in terms of study design and population enrollment. Males were more represented than females in most studies (68.8%, 11/16). The median (range) age in all studies were 37 (33–41). The median (range) CD4 T cells count in all studies was 180(49–402). Five studies indicated that more than half patients were under antiretroviral therapy while patients in 3 studies were not. Seven (43.8%) studies included only susceptive TB cases and 4 (25%) included patients regardless of symptoms while 5 (31.3%) research conducted on pulmonary TB individuals.

### Quality of the selected studies

The quality of the selected studies was very high as evaluated by the QUADAS tool. Study quality indicators were met by 100% (14/14 items in QUADAS tool) in 11 (68.8%) studies and 93% (13/14 items) in other 5 (31.3%) researches, thereby increasing the strength of scientific evidence of our review.

### Sensitivity

**Figure 2:**
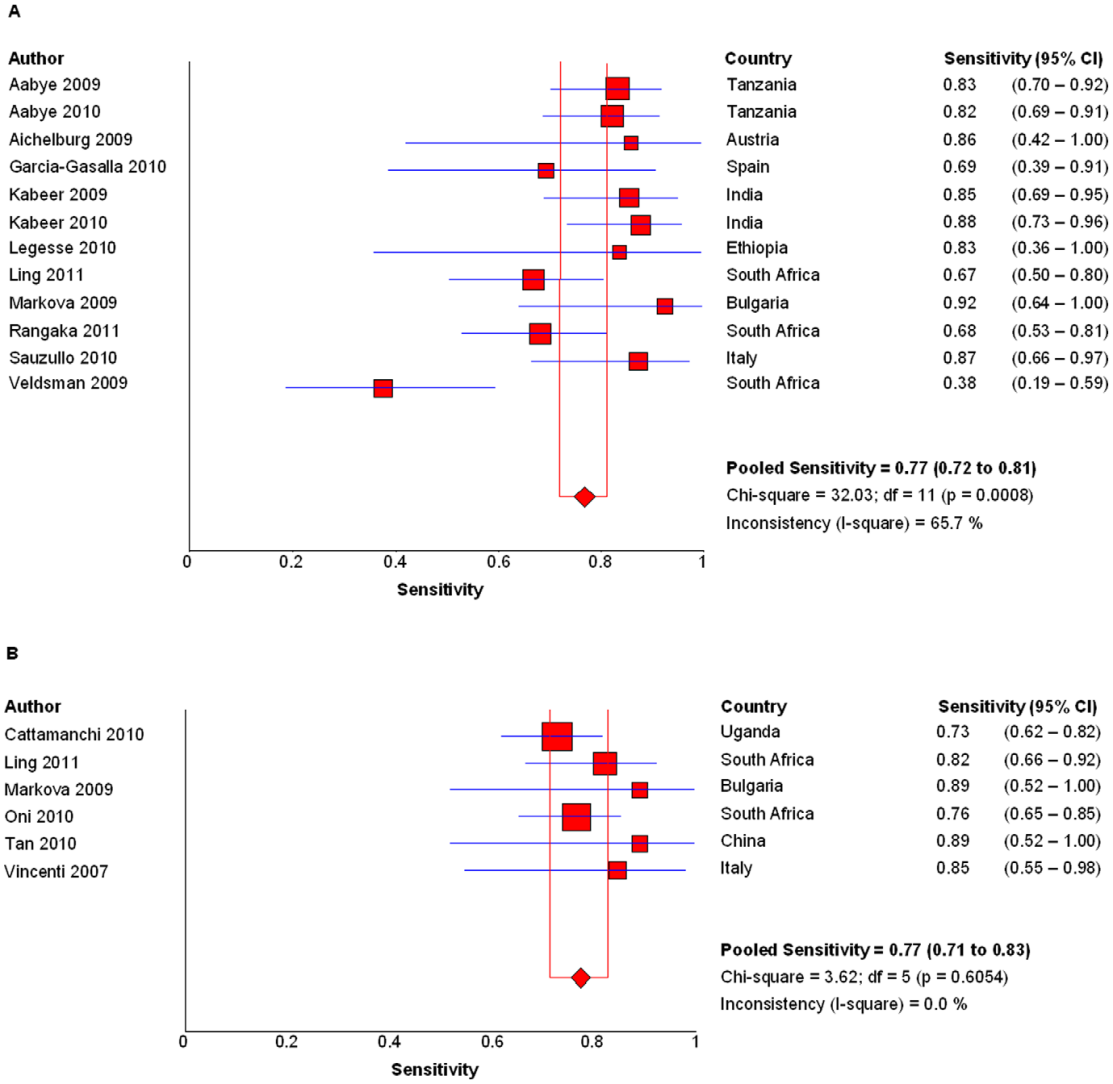
Sensitivities of IGRAs. Forest plots of pooled sensitivity of QFT QFT-GIT (A) and T-SPOT (B). Date were calcaulated based on all studies that reported sensitivity of IGRAs after excluding indeterminate results.

After excluding the indeterminate results, the pooled sensitivity of QFT-GIT was 76.7% (95%CI, 71.6–80.5%, I^2^ = 63.3%, *P* = 0.001, fig. 2A). QFT-GIT conducted in high-income countries (81.4%; 95%CI, 66.6–91.6%; I^2^ = 0%, *P* = 0.42) showed slightly higher sensitivity then that in low/middle-income countries (76.1%; 95%CI, 70.9–80.7%; I^2^ = 73.1%, *P*<0.001). The pooled sensitivity of T-SPOT was 77.4% (95%CI, 71.4–82.6%; I^2^ = 0%, *P* = 0.61, fig. 2B). The sensitivity was 77% (95%CI, 70.1–82%; I^2^ = 0%, *P* = 0.51) in low/middle-income countries while the only study from high-income country showed a sensitivity of 84.6% (95%CI, 54.6–98.1%). The sensitivity of both IGRAs decreased significantly if indeterminate results were deemed negative. It was 68.7% (95%CI, 62–71.4%; I^2^ = 71.4%, *P*<0.001) for QFT-GIT and 65.7% (95%CI, 59.7–71.3%; I^2^ = 69.6%, *P*<0.01) for T-SPOT respectively.

### Specificity

**Figure 3:**
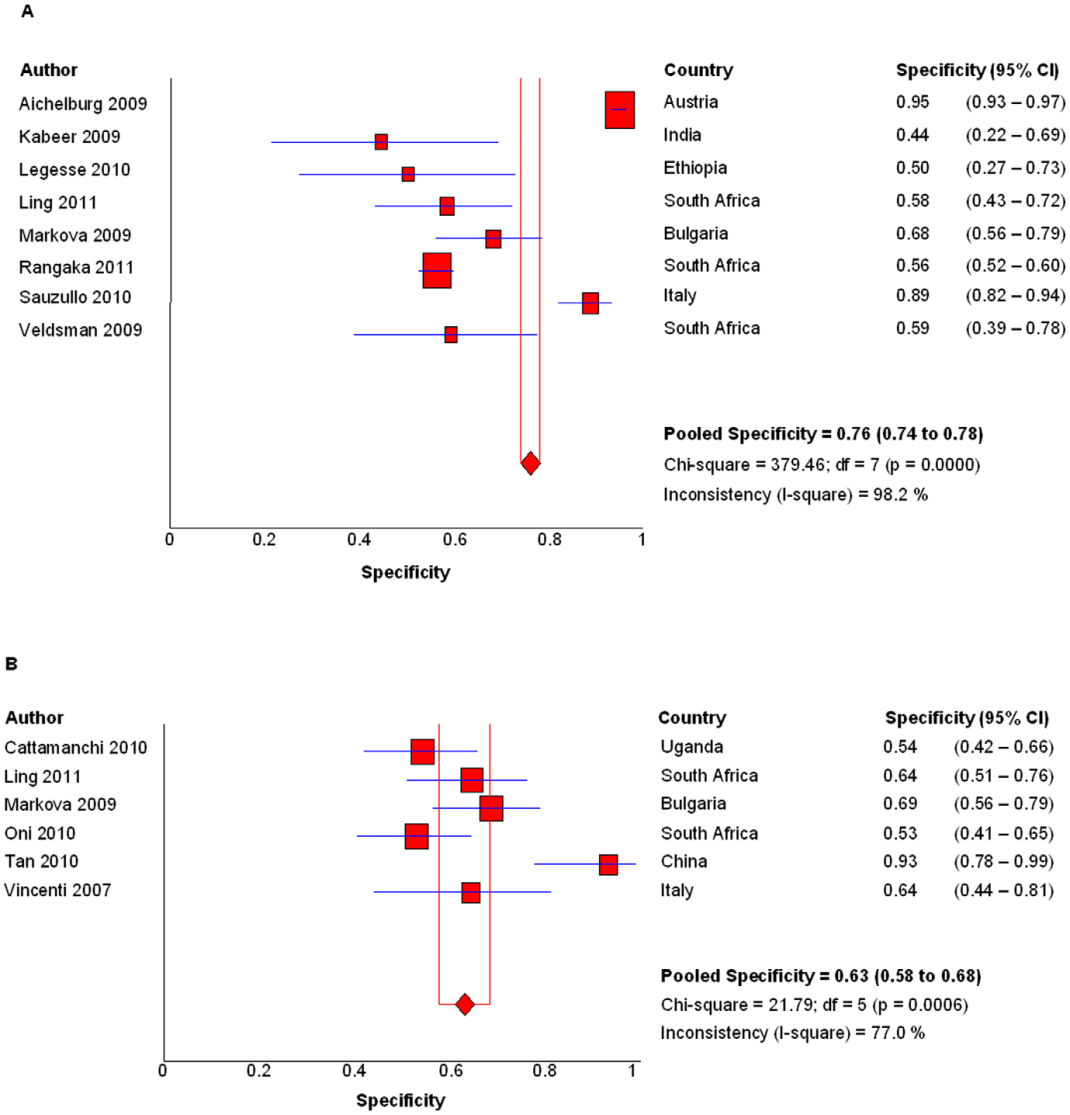
Specificities of IGRAs. Forest plots of pooled specificity of QFT-GIT (A) and T-SPOT (B). Date were calcaulated based on all studies that reported specificity of IGRAs after excluding indeterminate results.

Four studies enrolled only active TB patients were excluded for specificity calculation. The pooled specificity of QFT-GIT was 76.1% (95%CI, 74.0–78.0%; I^2^ = 98.2%, *P*<0.001; fig. 3A). The specificity from studies conducted in high-income country was 94.2% (95%CI, 92.5–95.6%; I^2^ = 85.5%, *P*<0.01) when it was just 57% (53.7–60.4%; I^2^ = 8.9%, *P* = 0.36) from research done in low/middle-income countries. The pooled specificity of T-SPOT was 63.1% (95%CI, 57.6–68.3%, I^2^ = 77%, *P* = 0.001; fig. 3B) from all studies. It was 63% (95%CI, 57.3–68.4%; I^2^ = 82%, *P*<0.001) in low/middle-income countries while the only study from high-income country showed a specificity of 64.3% (95%CI, 44.1–81.4%).

### Indeterminate results

**Figure 4:**
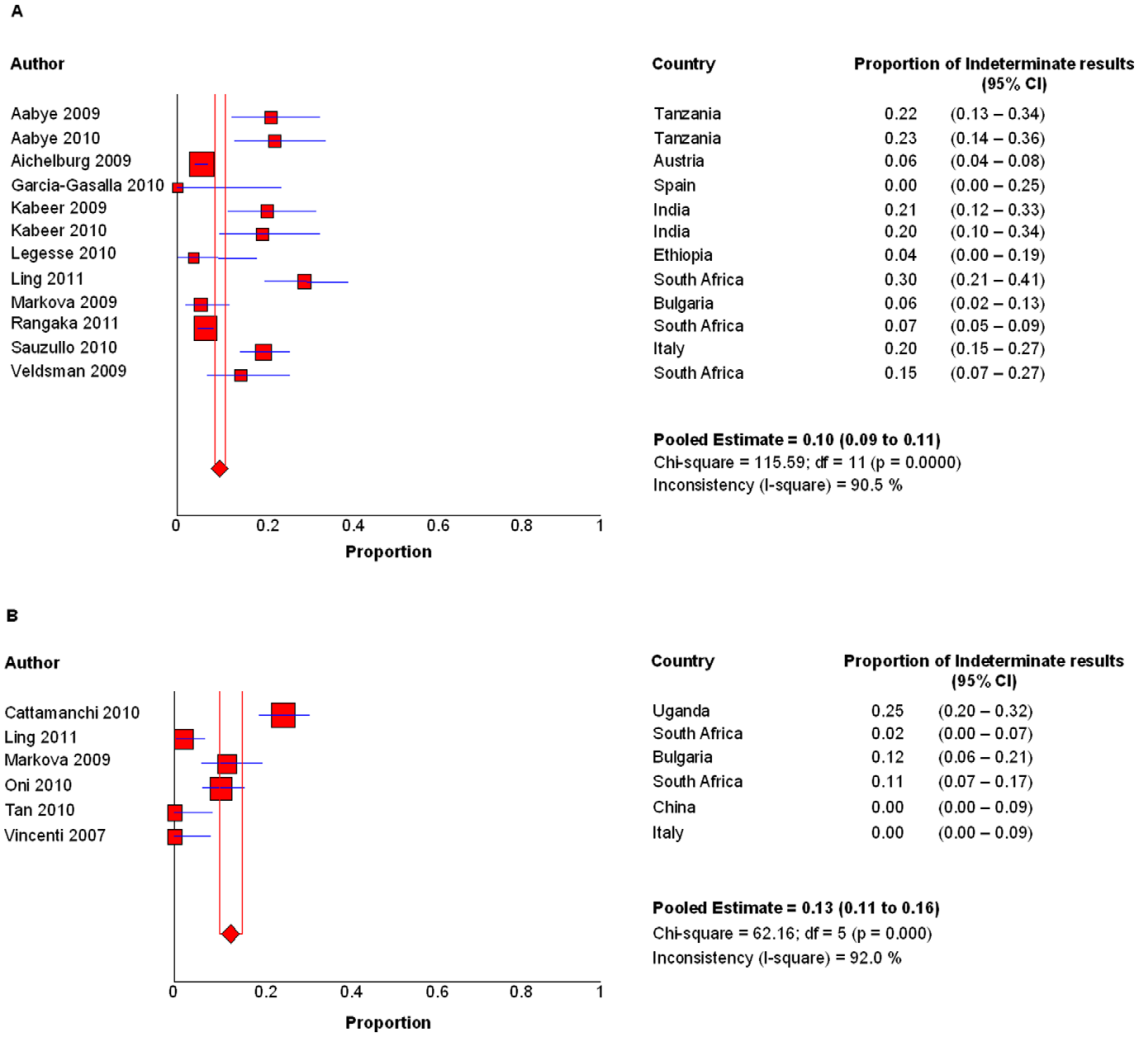
Proportion of indeterminate results of IGRAs. Forest plots of pooled proportion of indeterminate resluts of QFT-GIT (A) and T-SPOT (B).

The pooled proportion of indeterminate results of QFT-GIT and T-SPOT were 10% (95%CI, 8.8–11.3%) and 13.2% (95%CI, 10.6–16.0%), respectively, as showed in fig. 4. They were slightly higher when conducted in low/middle-income countries (11.4%; 95%CI,9.7–13.2% and 14.0%; 95%CI 11.4–17.1% for QFT-GIT and T-SPOT, respectively) comparing to that in high-income countries (8.4%; 95%CI, 6.8–10.2% and 0%; 95%CI, 0–0.86% for QFT-GIT and T-SPOT, respectively).

## Discussion

Early diagnosis of the active TB is especially needed in HIV infected cases, since there is an accelerated progression of TB and higher mortality. Meantime, ruling out active TB in HIV infected patients prior to isoniazid preventive therapy (IPT) is also necessary. However, besides *M.tuberculosis* culture, there are only limited methods to diagnose active TB with poor accuracy in HIV-infected patients, which urge the development of alternative, rapid and accurate method. In this study, we found that IGRAs are neither sensitive enough to rule out active TB nor specify to distinguish latent TB infection and active TB in HIV-infected patients.

The sensitivities of both IGRAs in diagnosis of active TB increased after excluded indeterminate results in HIV-infected patients. However, they were still not sensitive enough to rule out active TB alone as they missed more than 20% patients. A recent meta-analysis by Cattamanchi and colleagues showed the sensitivities of QFT-GIT and T-SPOT in diagnosis of latent TB infection in HIV-infected patients were 61% and 72% in low/middle-income countries (indeterminate results included), respectively when using active TB as a surrogate reference standard [31]. Another meta-analysis from Hoffmann and Ravn showed pooled sensitivities of 79% and 80.5% for QFT-GIT and T-SPOT in diagnosis of active TB after excluded indeterminate results respectively [32]. The slight differences between our results and theirs may be mainly because they also enrolled studies that active TB cases were not confirmed by culture and results were not interpreted according to manufacturer-recommended cut-off value. The sensitivities of both IGRAs were higher in a recent meta-analysis performed on general patients (a few studies also included children and immunosuppressed patients; 81% and 87.5% in their meta-analysis versus 76.7% and 77.4% in ours for QFT-GIT and T-SPOT, respectively after excluded indeterminate results) [33]. Thus, HIV infection may impact the performance of IGRAs, which is consistent with results from others [17], [23], [34]. However, Tsiouris and colleagues showed no impact of HIV infection on IGRAs sensitivity which might be explained by high proportion of active TB cases that under treatment [35].

The pooled specificities of QFT-GIT, T-SPOT in this analysis were as low as 75.9%, and 63.1%, respectively, indicating the poor ability of IGRAs in distinguishing latent TB infection and active TB. Apparently, the low specificity is due to the high latent TB infection rate in our selected studies. This is mainly because the most studies we enrolled were conducted in low/middle-income countries with a high TB burden. The two studies from Italy and Austria using QFT-GIT did showed relatively higher specificities. However, the specificity of T-SPOT was also low in the trail completed in Italy by Vincenti and colleagues. This may be due to that nearly one third of non-active TB patients in this study were from low/middle-income countries (i.e. South American and African). Thus, IGRAs may potentially have an adjunctive role in ruling in active TB in HIV-infected patients in high-income countries, but it still needs further investigation.

The high proportion of indeterminate results of IGRAs in HIV-infected patients further dampened their application. In current meta-analysis, the high proportion of indeterminate results could be mainly because of the high level of immunosuppression. Cattamanchi and colleagues showed that the pooled proportion of indeterminate results was significantly higher when CD4 T cell count was less than 200 cells/mL versus greater than 200 cells/mL for QFT-GIT but not T-SPOT in HIV-infected patients [31]. However, technical error might also be a cause. The procedure of T-SPOT test is relatively more technically-demanding than QFT-GIT, partially explaining the relatively higher indeterminate results of T-SPOT in this meta-analysis.

There are some limitations in our meta-analysis. Due to our strict inclusion/exclusion criteria, most studies (or the number of eligible individuals) were small, especially in terms of the number of confirmed active TB cases. In addition, number of studies conducted in high-income countries was limited.

In conclusion, the current evidence brought forward in this systematic review and meta-analysis shows that the IGRAs in their current formulations have limited accuracy in diagnosing active TB in HIV-infected patients, and should not be used alone to rule out or rule in active TB cases in HIV-infected patients. Further modification is needed to improve their accuracy.

## Supporting Information

Checklist S1 PRISMA checklist of the meta-analysis.
(DOC)

Protocol S1 Protocol of the meta-analysis.
(DOC)

Flow Diagram S1 Flow diagram for study selection.
(DOC)
