# Supplementary material for: Interferon-Gamma Release Assays for the Diagnosis of Active Tuberculosis in HIV-Infected Patients: A Systematic Review and Meta-Analysis
Source: PLoS One. 2011 Nov 1;6(11):e26827. doi: 10.1371/journal.pone.0026827 (PMC3206065; doi:10.1371/journal.pone.0026827)
Supplement: Protocol S1 — Protocol of the meta-analysis. (DOC) [file pone.0026827.s002.doc]

**Title:** Interferon-gamma release assays for the diagnosis of active tuberculosis in HIV-infected patients: a systematic review and meta-analysis

**Abstract**

**Background:** Interferon-gamma release assays (IGRAs) has provided a new method for the diagnosis of *Mycobacterium tuberculosis* infection. However, the role of IGRAs for the diagnosis of active tuberculosis (TB), especially in HIV-infected patients remains unclear. **Methods:** We will search PubMed, EMBASE and Cochrane databases to identify studies published in January 2001till now that evaluated the evidence of using QuantiFERON-TB Gold in-tube (QFT-GIT) and T-SPOT.TB (T-SPOT) on blood for the diagnosis of active TB in HIV-infected patients. Pooled sensitivities, specificities and proportion of indeterminate results of IGRAs will be cacluated.

**Objective:** To evaluate the accuracy of interferon gamma release assay in diagnosis of active TB among HIV-infected patients.

**Types of study to be included**

No restrictions of study design.

**Condition or domain being studied**

Tuberculosis in HIV-infected patients. Diagnosis of active tuberculosis is important for both therapy and prevention.

**Participants/ population**

HIV-infected patients who undergo active TB detection using two commercially available interferon gamma release assays, QuantiFERON-TB Gold In-Tube ([QFT-GIT]; Cellestis, Carnegie, Australia) or T-SPOT.TB ([TSPOT]; Oxford Immunotec, Abingdon, UK) performed on blood sample.

**Diagnosis methods**

Two commercially available interferon gamma release assays, QuantiFERON-TB Gold In-Tube or T-SPOT.TB performed on blood sample.

**Reference Diagnosis methods**

Specimen Mycobacterium. tuberculosis culture or characteristic histopathological findings

**Outcome(s)**

***Primary outcomes***

Sensitivity and specificity of QuantiFERON-TB Gold In-Tube or T-SPOT.TB in diagnosis of active TB in HIV-infected patients

***Secondary outcomes***

Proportion of indeterminate results of QuantiFERON-TB Gold In-Tube or T-SPOT.TB

**Search strategy**

Electronic searches of abstracts and titles will be conducted in PubMed and Embase. The Cochrane Central Register of Controlled Trials. We also plan to contact the authors for detail information.

**Data extraction, (selection and coding)**

Two researchers independently will select eligible publications and will extract data on the included studies, with disagreements resolved by consensus. Search results will be imported to EndNote and duplicated resulted will be deleted.

**Risk of bias (quality) assessment**

Two investigators will independently assess methodological study quality by using the QUADAS (Quality Assessment of Diagnostic Accuracy Studies) tool. The interobserver agreement in the QUADAS assessment will be determined by using Cohen's statistics. Publication bias will be examined by construction of a funnel plot.

**Strategy for data synthesis**

For each study, 2 x 2 contingency tables will be constructed. Pooled sensitivity, specificity will be calculated using bivariate models with a random-effects approach. The analysis will be performed using the Meta-DiSc and Stata software.

**Start date:** 06 May 2011
