# Supplementary material for: Interferon-Gamma Release Assays for the Diagnosis of Active Tuberculosis in HIV-Infected Patients: A Systematic Review and Meta-Analysis
Source: PLoS One. 2011 Nov 1;6(11):e26827. doi: 10.1371/journal.pone.0026827 (PMC3206065; doi:10.1371/journal.pone.0026827)
Supplement: Flow Diagram S1 — Flow diagram for study selection. (DOC) [file pone.0026827.s003.doc]

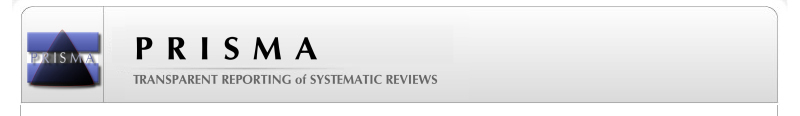
**PRISMA 2009 Flow Diagram**

**Screening**

**Included**

**Eligibility**

**Identification**

Records identified through database searching
(n =621)

Records after duplicates removed
(n = 590 )

Records screened
(n =590 )

Records excluded
(n = 555 )

Full-text articles assessed for eligibility
(n = 35 )

Full-text articles excluded (n = 19)

Reasons:

Older generation IGRAs: 4

Active TB cases were not culture confirmed: 5

Cut-off not same as manufacture’s recommendation: 2

Less than 10 HIV-infected patients: 2

Patients under anti-tuberculosis therapy: 2

Not commercial IGRAs: 2

Repeat study: 1

Study on children: 1

Studies included in meta-analysis

(n = 16 )
